# New Briarane Diterpenoids from Taiwanese Soft Coral *Briareum violacea*

**DOI:** 10.3390/md12084677

**Published:** 2014-08-22

**Authors:** Chia-Ching Liaw, Yuan-Bin Cheng, Yun-Sheng Lin, Yao-Haur Kuo, Tsong-Long Hwang, Ya-Ching Shen

**Affiliations:** 1 School of Pharmacy, College of Medicine, National Taiwan University, Taipei 100, Taiwan; E-Mails: biogodas@hotmail.com (C.-C.L.); jmb@kmu.edu.tw (Y.-B.C.); x00010106@meiho.edu.tw (Y.-S.L.); 2 Department of Marine Biotechnology and Resources, National Sun Yat-Sen University, Kaohsiung 804, Taiwan; 3 Graduate Institute of Natural Products, School of Pharmacy, Kaohsiung Medical University, Kaohsiung 807, Taiwan; 4 Department of Biological Science & Technology, Mei Ho University, Pingtung 912, Taiwan; 5 Division of Chinese Materia Medica Development, National Research Institute of Chinese Medicine, Taipei 112, Taiwan; E-Mail: kuoyh@nricm.edu.tw; 6 Graduate Institute of Natural Products, Chang Gung University, Taoyuan 333, Taiwan; E-Mail: htl@mail.cgu.edu.tw

**Keywords:** *Briareum violacea*, briarane diterpenoids, briaviolides, anti-inflammatory activities

## Abstract

Ten new briarane diterpenoids, briaviolides A–J (**1**–**10**), together with six known briaranes, solenolides A and D, excavatolide A, briaexcavatolide I, 4β-acetoxy-9-deacetystylatulide lactone and 9-deacetylstylatulide lactone, were isolated from the Taiwanese soft coral, *Briareum violacea*. Their structures were determined on the basis of spectroscopic data (^1^H- and ^13^C-NMR, ^1^H–^1^H COSY, HSQC, HMBC and NOESY), HR-MS and chemical methods. The absolute configuration of briaviolide A (**1**) was determined by X-ray crystallographic analysis. Compounds **5**, **9** and derivative **11** showed moderate inhibitory activities on superoxide-anion generation and elastase release by human neutrophils in response to *N*-formyl-methionyl-leucyl-phenylalanine/Cytochalasin B (fMLP/CB).

## 1. Introduction

The briarane diterpenoids [1] continue to attract the attention of natural product chemists because of their structural complexity and interesting biological activities, such as anti-inflammatory [2], antiviral [3], cytotoxic [4,5,6], antifouling [7,8], immuno-modulatory [9], insecticidal [10] and reversal of multidrug resistance [11]. The structures of these diterpenoids are characterized by a highly oxygenated bicyclo[8.4.0]tetradecane skeleton that is frequently attached with a γ-lactone moiety. Since the first structural elucidation of briarein A isolated from *Briareum asbestinum* in 1977 [12], more than 450 briarane-type diterpenoids have been reported from Octocorallia, including Gorgonacea, Pennatulacea, Alcyonacea and Stolonifera [13,14,15]. In serial studies of the Taiwanese gorgonian corals, many new briaranes have been isolated, including juncenolides A–G from *Junceella juncea* [16,17,18], frajunolides A–K from *J. fragilis* [19,20] and briaviodiol A from *B. violacea* [21].

**Figure 1:**
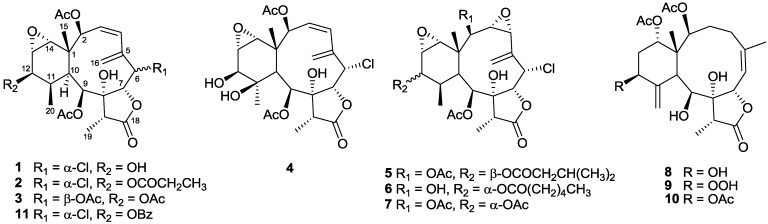
Briaviolides A–J (**1**–**10**) isolated from *Briareum violacea*.

In this paper, we report the investigation of Taiwanese soft coral *Briareum violacea* that provided ten new briarane-type diterpenoids, briaviolides A–J (**1**–**10**) (Figure 1), along with six known analogues, solenolides A and D, excavatolide A, briaexcavatolide I, 4β-acetoxy-9-deacetystylatulide lactone and 9-deacetylstylatulide lactone. The structures of new compounds were established by spectroscopic and chemical methods. Among them, the structure of **1** was further confirmed by single-crystal X-ray analysis. The *in vitro* anti-inflammatory activities of new compounds (**1**–**10**) and new derivative **11** were also tested for their inhibition of elastase release and superoxide-anion generation from human neutrophils.

## 2. Results and Discussion

**Figure 2:**
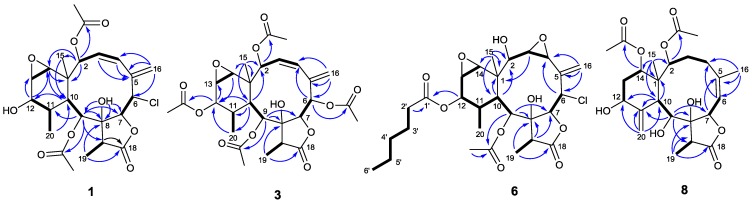
Key HMBC (arrows) and COSY (bold lines) correlations of **1**, **3**, **6** and **8**.

Briaviolide A (**1**) was isolated as colorless prisms. Its ESIMS revealed two isotopic [M + Na] ^+^ and [M + Na + 2]^+^ peaks for *pseudo*-molecular ions at *m/z* 521 and 523 (3:1) and HRESIMS at *m/z* 521.1554 [M + Na]^+^, indicating a molecular formula C_24_H_31_O_9_Cl, which contains one chlorine atom and accounts for nine degrees of unsaturation. The IR spectrum of **1** showed absorption frequencies at 3524, 1767 and 1737 cm^−1^, indicating the presence of hydroxyl, γ-lactone and carbonyl ester functionalities, respectively. The ^1^H- and ^13^C-NMR spectroscopic data (Table 1 and Table 2) exhibited signals of two acetate methyl singlets at δ_H_ 2.09 (δ_C_ 21.1) and 2.17 (δ_C_ 22.1), with corresponding carbonyl signals at δ_C_ 170.3, 170.2, respectively. The carbonyls showed respective HMBC connectivity (Figure 2) with two methine doublets at δ_H_ 6.09 and 5.30, revealing the positions of each acetate group (C-2 and C-9, respectively). The carbonyl signal of a γ-lactone (δ_C_ 174.9) was connected with a secondary methyl doublet at δ_H_ 1.17 (δ_C_ 6.3) by the HMBC (H-19/C-18) and COSY (H-19/H-17) correlations. The connection between C-17 and C-8 (δ_C_ 83.9) was elucidated by the HMBC correlation from H-19 to C-8. The hydroxyl proton (δ_H_ 3.52) of C-8 was correlated with the adjacent lactonide carbon C-7 and C-9 by virtue of the HMBC correlations from 8-OH to C-7 and C-9. These correlations along with the H-6/H-7 (COSY) correlation fixed the position of the lactone ring. The signals of an exocyclic double bond were observed at δ_H_ 6.24 (br s) and 5.95 (d, *J* = 2.1 Hz) (δ_C_ 118.7) and were conjugated with an endocyclic double bond at δ_H_ 5.63 (dd, *J* = 11.4, 9.0 Hz; δ_C_ 131.0), 5.90 (d, *J* = 11.4 Hz; δ_C_ 128.0) by related HMBC and COSY correlations. The correlations of H-6/H-7 (COSY) and H-16/C-6 (HMBC) thus connected the conjugated double bond with the γ-lactone ring. The signals of a pair of methine protons at δ_H_ 3.23 (H-13) and δ_H_ 2.99 (H-14) were assigned for an epoxide ring. This moiety was neighbored on a quaternary carbon (δ_C_ 40.6) and a hydroxylated carbon (δ_C_ 70.5; δ_H_ 3.71) by the HMBC correlation of H-12/C-13 and H-14/C-1. The COSY correlations of H-12/H-11/H-20 and H-9/H-10 indicated the presence of two proton sequences. These two sequences were connected by the HMBC correlations of H-20/C-10 and H-9/C-11. By deducing the unsaturation of two acetates, conjugated diene, a γ-lactone ring and the epoxide ring, the remaining two degrees of unsaturation strongly suggested that Compound **1** possesses two additional rings belong to briarane-type diterpenoids.

A literature survey revealed that the ^1^H- and ^13^C-NMR spectroscopic data of **1** showed similarity with those of briaexcavatolide I [22]. The only difference between them was **1** having two acetyl groups, while there are three in briaexcavatolide I. Acetylation of **1** gave a triacetyl product that was identical to briaexcavatolide I after comparing with their ^1^H and ^13^C NMR data. Benzoylation of **1** yielded a monobenzoyl derivative **11** that confirmed the secondary hydroxyl group at C-12.

**Figure 3:**
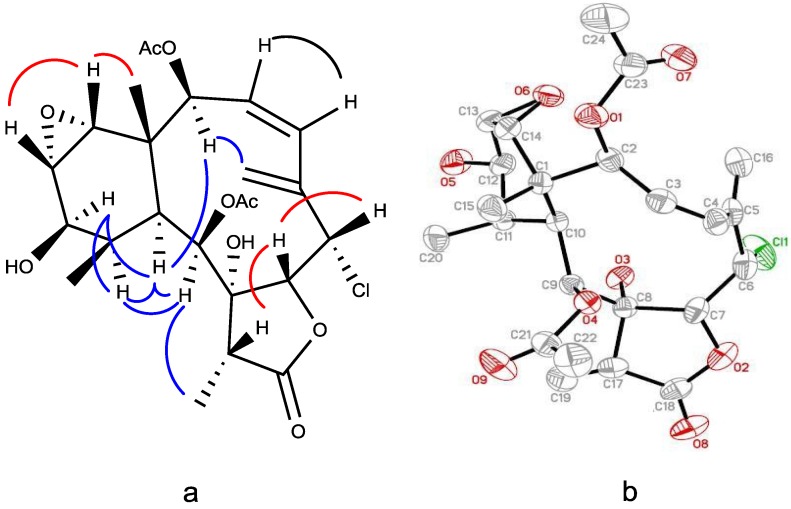
(**a**) Key NOESY correlations of Compound **1**; (**b**) ORTEP (Oak Ridge Thermal Ellipsoid Plot) diagram showing the crystallographic atom-numbering scheme and solid state conformation of **1**.

The relative configuration of **1** was determined by NOESY (Figure 3a) and X-ray diffraction analysis (Figure 3b). Naturally occurring briaranes have β-face of Me-15 and α-orientation of H-10. The NOESY correlations between H-2/H-16, H-2/H-10, H-10/H-12, H-12/H-11, H-11/H-9 and H-9/Me-19 required that all of these groups were in α-face, and correlations of H-6/H-7, H-7/H-17, Me-15/H-14 and H-14/H-13 indicated β-disposition for these groups. The correlations between H-16/H-2 and H-2/H-10 suggested that the conjugated diene had a *s-cis* geometry and forced the ten-membered ring to adopt a boat-like conformation with C-1 and C-5 at the bow and stern positions. An X-ray crystallographic analysis established the complete structure and stereochemistry of **1** as shown by the Oak Ridge Thermal Ellipsoid Plot (ORTEP) diagram stereo-drawing in Figure 3b. The negative optical rotation value of **1** was similar to that of briaexcavatolide I [23] in direction and magnitude, suggesting that **1** and briaexcavatolide I had 1*S*,10*S*-configurations in the ring junction. Thus, the structure of briaviolide A (**1**) was determined as (1*S*,2*S*,3*Z*,6*S*,7*R*,8*R*,9*S*,10*S*,11*R*,12*R*,13*S*,14*R*,17*R*)-6-chloro-13,14-epoxy-2,9-diacetoxy-8,12-dihydroxybriaran-3(4),5(16)-dien-18,7-olide.

**Table 1:** ^1^H-NMR spectroscopic data (in ppm, *J* in Hz) of briaviolides A–J (**1**–**10**) and Compound **11**.

No.	1	2	3	4	5	6	7	8 ^a^	9 ^b^	10 ^c^	11
2	6.09 (d, 9.0)	6.12 (d, 9.2)	6.10 (d, 9.5)	6.21 (d, 10.0)	5.13 (d, 9.3)	3.66 (d, 8.0)	5.23 (d, 9.5)	5.34 (d, 9.2)	5.16 (d, 7.6)	5.76 (d, 8.8)	6.19 (d, 9.0)
3	5.63 (dd, 11.4, 9.0)	5.16 (dd, 11.2, 9.2)	5.61 (dd, 11.5, 9.5)	5.60 (dd, 11.6, 9.6)	3.39 (dd, 9.3, 3.9)	3.39 (dd, 8.0, 3.6)	3.41 (dd, 9.5, 4.4)	2.90 (m)	2.90 (td, 14.8, 5.2)	3.32 (m)	5.65 (dd, 12.0, 9.0)
								1.45 (m)	1.49 (m)	1.67 (m)	
4	5.90 (d, 11.4)	5.89 (d, 11.2)	6.15 (d, 11.5)	5.93 (d, 10.0)	3.60 (d, 4.0)	3.72 (d, 3.6)	3.66 (d, 4.0)	2.47 (br d, 10.0)	2.48 (br d, 14.0)	2.53 (br d, 15.6)	5.93 (d, 12.0)
								1.73 (td, 15.2, 4.4)	1.83 (dd, 14.0, 4.4)	1.80 (m)	
6	5.21 (br s)	5.20 (br s)	5.75 (d, 10.0)	5.08 (br d, 2.8)	5.37 (d, 2.0)	5.40 (br s)	5.43 (d, 3.5)	5.31 (d, 11.6)	5.41 (br d, 9.6)	5.88 (d, 8.8)	5.23 (br s)
7	4.94 (d, 3.6)	4.93 (d, 3.2)	4.66 (d, 10.0)	4.97 (d, 4.4)	5.03 (d, 2.0)	4.68 (br s)	5.02 (d, 3.0)	5.52 (d, 9.6)	5.51 (d, 10.0)	6.01 (d, 9.6)	4.79 (d, 3.6)
9	5.30 (d, 7.8)	5.24 (d, 7.6)	5.29 (d, 8.0)	5.76 (d, 6.0)	5.32 (m)	5.30 (d, 9.0)	5.33 (d, 9.0)	4.68 (br s)	4.40 (m)	5.10 (m)	5.30 (d, 7.5)
10	1.95 (dd, 7.8, 2.0)	2.01 (dd, 7.2, 2.0)	2.03 (m)	2.22 (d, 6.0)	1.74 (dd, 8.8, 2.4)	1.83 (m)	2.04 (dd, 9.0, 2.5)	3.24 (s)	3.18 (br s)	3.89 (s)	1.84 (d, 7.2)
11	2.07 (m)	2.05 (m)	2.22 (m)		2.30 (m)	2.30 (m)	2.35 (m)				2.10 (m)
12	3.71 (d, 4.2)	4.62 (d, 4.4)	4.63 (d, 5.0)	3.56 (s)	4.64 (d, 4.8)	4.68 (br s)	4.70 (dd, 5.0, 2.5)	4.13 (dd, 10.8, 5.2)	4.40 (m)	5.50 (m)	4.99 (d, 4.5)
13	3.23 (d, 3.3)	3.15 (d, 3.2)	3.18 (br d, 1.5)	3.24 (d, 4.0)	3.12 (br s)	3.60 (m)	3.53 (dd, 3.5, 5.5)	2.01 (m)	2.09 (ddd, 14.4, 6.0, 3.2)	2.38 (m)	3.32 (d, 3.0)
								1.50 (m)	1.67 (ddd, 14.4, 9.2, 3.2)	1.86 (m)	
14	2.99 (d, 3.6)	2.97 (d, 3.6)	2.99 (d, 3.0)	2.94 (d, 4.0)	2.90 (d, 3.6)	3.24 (d, 3.0)	2.87 (d, 3.0)	4.68 (br s)	4.73 (t, 3.2)	5.10 (m)	3.04 (d 3.3)
15	1.13 (s)	1.12 (s)	1.14 (s)	1.21 (s)	1.23 (s)	1.11 (s)	1.19 (s)	1.08 (s)	1.17 (s)	1.42 (s)	1.17 (s)
16	6.24 (br s)	6.26 (br s)	6.10 (s)	6.23 (br s)	6.13 (d, 2.0)	5.61 (br s)	6.11 (d, 2.5)	1.85 (s)	1.91 (s)	2.04 (s)	6.31 (br s)
	5.95 (d, 2.1)	6.00 (s)	5.72 (s)	5.94 (s)	6.04 (d, 2.0)	5.98 (d, 1.8)	6.07 (d, 2.5)				6.04 (d, 1.8)
17	2.37 (q, 6.9)	2.35 (q, 7.6)	2.48 (q, 7.0)	2.37 (q, 7.6)	2.45 (q, 6.9)	2.43 (q, 7.2)	2.41 (q, 7.0)	3.05 (q, 7.2)	3.09 (q, 7.2)	3.63 (q, 7.6)	2.34 (q, 7.5)
19	1.17 (d, 6.9)	1.14 (d, 7.6)	1.16 (d, 7.0)	1.18 (d, 7.6)	1.18 (d, 6.9)	1.16 (d, 7.2)	1.19 (d, 6.5)	1.02 (d, 7.2)	1.06 (d, 7.2)	1.43 (d, 7.6)	1.18 (d, 7.5)
20	1.04 (d, 6.9)	1.05 (d, 6.8)	1.05 (d, 7.0)	1.29 (s)	1.03 (d, 6.9)	1.05 (d, 7.2)	1.06 (d, 7.5)	5.45 (s)	5.32 (br s)	5.65 (s)	1.16 (d, 7.2)
								5.22 (s)	5.25 (br s)	5.48 (s)	
2'		2.38 (m)			2.20 (m)	2.28 (m)					
3'		1.15 (t, 8.0)			2.10 (m)	1.61 (m)					7.46 (m)
4'					0.96 (d, 6.3)	1.31 (m)					7.59 (t, 7.2)
5'					0.96 (d, 6.3)	1.31 (m)					7.47 (m)
6'						0.90 (t, 7.0)					7.59 (t, 7.2)
7'											7.46 (m)
2-OAc	2.09 (s)	2.07 (s)	2.08 (s)	2.18 (s)	2.12 (s)	2.20 (s)	2.14 (s)	1.90 (s)	1.96 (s)	2.06 (s)	2.09 (s)
6-OAc			2.07 (s)								
9-OAc	2.17 (s)	2.15 (s)	2.13 (s)	2.08 (s)	2.19 (s)	2.20 (s)	2.22 (s)				2.19 (s)
12-OAc			2.19 (s)				2.06 (s)			2.05 (s)	
14-OAc								1.90 (s)	1.94 (s)	1.85 (s)	
8-OH	3.52 (s)		3.48 (s)							7.38 (s)	3.49 (s)
9-OH										7.79 (d, 7.6)	

^a^ Recorded in *d_6_*-acetone at 400 MHz; ^b^ Recorded in pyridine-*d*_5_ at 400 MHz; ^c^ Recorded in CD_3_OD at 400 MHz.

**Table 2:** ^13^C-NMR spectroscopic data (δ in ppm, mult.) of briaviolides A–J (**1**–**10**) and Compound **11** ^a^.

No.	1	2	3	4	5	6	7	8 ^b^	9 ^c^	10 ^d^	11
1	40.6 (s)	41.9 (s)	40.8 (s)	42.9 (s)	38.3 (s)	38.8 (s)	38.5 (s)	49.5 (s)	48.9 (s)	47.7 (s)	41.9 (s)
2	75.5 (d)	75.8 (d)	75.3 (d)	75.6 (d)	74.9 (d)	74.0 (d)	75.5 (d)	74.8 (d)	76.6 (d)	73.4 (d)	76.0 (d)
3	131.0 (d)	130.4 (d)	130.4 (d)	129.1 (d)	60.0 (d)	62.3 (d)	59.9 (d)	32.3 (t)	32.4 (t)	30.1 (t)	130.6 (d)
4	128.0 (d)	127.7 (d)	128.0 (d)	129.1 (d)	57.0 (d)	57.8 (d)	56.9 (d)	29.5 (t)	29.4 (t)	27.3 (t)	127.9 (d)
5	136.6 (s)	135.8 (s)	137.2 (s)	136.3 (s)	133.3 (s)	135.1 (s)	133.9 (s)	141.3 (s)	144.0 (s)	141.0 (s)	135.8 (s)
6	62.4 (d)	63.2 (d)	75.3 (d)	61.6 (d)	61.1 (d)	61.2 (d)	61.1 (d)	120.4 (d)	121.7 (d)	120.3 (d)	62.9 (d)
7	78.3 (d)	78.3 (d)	81.5 (d)	78.9 (d)	76.4 (d)	76.5 (d)	76.2 (d)	79.9 (d)	81.3 (d)	79.4 (d)	78.4 (d)
8	83.9 (s)	84.4 (s)	80.4 (s)	82.5 (s)	83.9 (s)	84.9 (s)	84.8 (s)	83.6 (s)	85.1 (s)	82.9 (s)	84.4 (s)
9	69.9 (d)	70.5 (d)	69.9 (d)	68.6 (d)	69.4 (d)	68.7 (d)	68.6 (d)	75.7 (d)	75.0 (d)	73.9 (d)	70.6 (d)
10	38.1 (d)	38.9 (d)	37.2 (d)	41.5 (d)	37.4 (d)	32.9 (d)	33.0 (d)	41.8 (d)	42.2 (d)	40.5 (d)	38.9 (d)
11	40.1 (d)	39.1 (d)	36.6 (d)	77.0 (d)	36.0 (d)	35.9 (d)	35.9 (d)	156.2 (s)	152.1 (s)	151.1 (s)	38.6 (d)
12	70.5 (d)	73.7 (d)	72.4 (d)	74.9 (d)	71.4 (d)	69.7 (d)	69.2 (d)	69.4 (d)	83.8 (d)	70.5 (d)	73.3 (d)
13	59.6 (d)	58.3 (d)	57.1 (d)	59.5 (d)	56.7 (d)	53.0 (d)	52.2 (d)	38.7 (t)	33.8 (t)	33.4 (t)	58.3 (d)
14	62.9 (d)	62.9 (d)	62.0 (d)	62.1 (d)	61.4 (d)	62.4 (d)	61.3 (d)	75.7 (d)	76.3 (d)	73.8 (d)	63.2 (d)
15	16.0 (q)	17.7 (q)	15.6 (q)	15.5 (q)	16.4 (q)	15.7 (q)	16.4 (q)	15.2 (q)	14.9 (q)	12.9 (q)	17.6 (q)
16	118.7 (d)	119.1 (d)	123.1 (d)	117.6 (d)	121.0 (d)	118.8 (d)	120.6 (d)	28.4 (q)	27.9 (q)	26.4 (q)	119.3 (d)
17	45.0 (d)	46.3 (d)	45.1 (d)	45.6 (d)	45.5 (d)	45.2 (d)	45.2 (d)	45.8 (d)	45.6 (d)	44.2 (d)	46.3 (d)
18	174.9 (s)	173.3 (s)	174.7 (s)	175.3 (s)	174.1 (s)	174.5 (s)	174.0 (s)	175.1 (s)	180.4 (s)	176.4 (s)	173.5 (s)
19	6.3 (q)	8.2 (q)	6.2 (q)	6.9 (q)	6.1 (q)	6.2 (q)	6.1 (q)	8.3 (q)	7.0 (q)	6.2 (q)	8.1 (q)
20	8.8 (q)	11.2 (q)	9.3 (q)	18.2 (q)	9.7 (q)	13.1 (q)	12.9 (q)	106.2 (t)	111.5 (t)	106.8 (t)	11.4 (q)
2-OCOCH_3_	170.3 (s)	169.0 (s)	169.7 (s)	170.2 (s)	169.0 (s)	169.8 (s)	170.0 (s)	168.6 (s)	172.3 (s)	169.4 (s)	169.3 (s)
2-OCOCH_3_	21.1 (q)	23.6 (q)	21.4 (q)	22.0 (q)	20.9 (q)	22.0 (q)	20.5 (q)	22.4 (q)	21.5 (q)	19.8 (q)	22.6 (q)
6-OCOCH_3_			169.6 (s)								
6-OCOCH_3_			21.0 (q)								
9-OCOCH_3_	170.2 (s)	168.7 (s)	170.2 (s)	169.8 (s)	169.5 (s)		169.4 (s)				168.9 (s)
9-OCOCH_3_	22.1 (q)	22.7 (q)	21.0 (q)	21.1 (q)	21.9 (q)		21.9 (q)				22.6 (q)
12-OCOCH_3_			170.3 (s)				169.2 (s)			168.0 (s)	
12-OCOCH_3_			21.9 (q)				20.9 (q)			19.5 (q)	
14-OCOCH_3_								169.0 (s)	172.7 (s)	168.9 (s)	
14-OCOCH_3_								22.1 (q)	21.3 (q)	19.8 (q)	
1′		171.2 (s)			171.9 (s)	173.1 (s)					164.7 (s)
2′		29.1 (t)			43.2 (t)	34.0 (t)					129.7 (s)
3′		10.9 (q)			25.7 (d)	24.8 (t)					129.5 (d)
4′					22.3 (q)	31.4 (t)					128.5 (d)
5′					22.3 (t)	22.4 (t)					133.0 (d)
6′						14.0 (q)					128.5 (d)
7′											129.5 (d)

^a^ Assignments made using the HSQC and HMBC techniques; ^b^ Recorded in Acetone*-d*_6_ at 100 MHz; ^c^ Recorded in pyridine-*d*_5_ at 100 MHz; ^d^ Recorded in CD_3_OD at 100 MHz.

Briaviolide B (**2**) was isolated as a colorless gum, having a molecular formula of C_27_H_35_O_10_Cl as deduced from the high-resolution ESIMS. Similar to those of **1**, Compound **2** showed IR bands at 3453, 1783, 1738 and 1732 cm^−1^, indicating hydroxyl, γ-lactone and ester carbonyl functionalities, respectively. Comparisons of its ^1^H- and ^13^C-NMR data (Table 1 and Table 2) with those of **1** revealed strong resemblance in all signals, except that the C-12 and H-12 signals in the NMR spectra of **2** were shifted downfield to δ_C_ 73.7 and δ_H_ 4.62 (d, *J* = 4.4 Hz), respectively, suggesting that Compound **2** had an ester group at position C-12. This ester group was revealed to be a propionyloxy group (δ_H_ 2.38, m; δ_H_ 1.15, t, *J* = 8.0 Hz). The structure of **2** was further supported by COSY, HSQC and HMBC experiments. The NOESY cross-peaks of **2** and **1** were quite similar, suggesting that they have the same relative configuration. Thus, briaviolide B (**2**) was established to be a 12-propionyloxy derivative of **1**.

**Figure 4:**
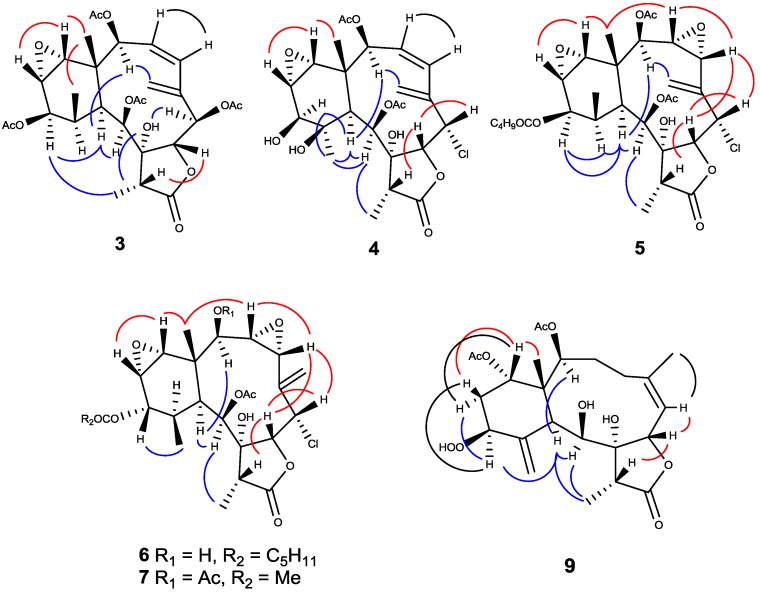
Key NOESY correlations of Compounds **3**–**7** and **9**.

The HRESIMS and ^13^C-NMR data (Table 2) of Compound **3** suggested a molecular formula of C_28_H_36_O_12_ that contains eleven degrees of unsaturation. It was found that the ^1^H-, ^13^C-NMR and IR spectroscopic data of **3** were very similar to those of Compound **1**, except for the signals of two more acetyl groups, including two methyl singlets, δ_H_ 2.07 (δ_C_ 21.0), δ_H_ 2.19 (δ_C_ 21.9), and the respective two carbonyls, δ_C_ 169.6, δ_C_ 170.3. The HMBC correlations (Figure 2) of H-6 (δ_H_ 5.75, d, *J* = 10.0 Hz)/δ_C_ 169.6 and H-12 (δ_H_ 4.63, d, *J* = 5.0 Hz)/δ_C_ 170.3 suggested that two acetoxyl group were attached at C-6 and C-12. Compound **3** was the first example of a briarane-type diterpenoid that contains an ester group at C-6. The configuration of Compound **3** was determined by using NOESY correlations (Figure 4) and comparing the data with those of **1**. The NOESY correlations of Me-15/Me-20, H-14; H-13/H-14 and H-7/H-17 indicated that all these atoms were β-oriented and the correlations of H-10/H-2, H-9, H-12; H-12/Me-19 suggested that all of these atoms were α-oriented. In addition, the NOESY correlations between OH-8 (δ_H_ 3.48, s) and H-6, H-10, Me-19 confirmed the *α*-orientation of H-6 and OH-8. On the basis of the above observations, the structure of briaviolide C (**3**) was assigned as the 2,6,9,12-tetraacetyl derivative of **1**, having 1*S*, 2*S*, 6*R*, 9*S*, 10*S*, 11*R*, 12*R*, 13*S* and 14*R* configurations.

A *pseudo*-molecular ion peak at *m/z* 537.1506 [M + Na]^+^ in the HRESIMS suggested that briaviolide D (**4**) had the molecular formula of C_24_H_31_O_10_Cl with nine degrees of unsaturation. The presence of a chlorine atom was supported by an isotope peak at *m/z* 539 in the LRESI spectrum, having one third of the intensity relative to *m/z* 537. The NMR spectroscopic data (Table 1 and Table 2) and IR spectrum revealed that Compound **4** possessed an 8-hydroxybriarane structure similar to that of Compound **1**, except for one additional hydroxyl group at C-11 (δ_C_ 77.0). This finding was confirmed from ^13^C-NMR data and HMBC correlations between H-10/C-11, H-12/C-11 and Me-20/C-11. The NOESY correlations of H-10/Me-11, H-12 required that the methyl group at C-11 be α-oriented. The configurations of other chiral centers are similar to Compound **1** as ascertained by NOESY experiments (Figure 4). Thus, the structure of briaviolide D (**4**) was established as (1*S*,2*S*,6*S*,7*R*,8*R*,9*S*,10*S*,11*R*,12*S*,13*S*,14*R*,17*R*)-6-chloro-13,14-epoxy-2,9-diacetoxy-8,11,12-trihydroxybriaran-3(4),5(16)-dien-18,7-olide.

The HRESIMS data of **5** agreed with the molecular formula C_29_H_39_O_11_Cl containing a chlorine atom and 10 degrees of unsaturation. The IR bands at 3429, 1779 and 1738 cm^−1^ suggested the presence of hydroxyl group, ester groups and a γ-lactone moiety. A detailed inspection of ^1^H and ^13^C-NMR spectroscopic data (Table 1 and Table 2) of **5** indicated the presence of an 8-hydroxybriarane skeleton with two epoxides, two acetate esters and an isovalerate ester group. It was found that the spectroscopic data of Compound **5** were very similar to those of brianolide [23]. However, cross comparisons of ^1^H- and ^13^C-NMR spectra showed that the 12-acetoxyl signals in brianolide were replaced by an isovalerate ester group (δ_H_ 2.20, m; 2.10, m; 0.96 d, 6H, *J* = 6.3 Hz); (δ_C_ 171.9, 43.2, 25.7, 22.3 × 2) in Compound **5**. The configurations of **5** was deduced by the NOESY analysis (Figure 4) and was based on the X-ray analysis of brianolide [24]. It was observed that the introduction of the 3,4-epoxide ring on the skeleton does not affect the boat-like conformation of the macro-ring, as revealed by the same NOESY interactions between H-16/H-2 and H-2/H-10 as those of Compound **1**. Thus, the structure of briaviolide E (**5**) was established as (1*R*,2*R*,3*R*,4*R*,6*S*,7*R*,8*R*,9*S*,10*S*,11*R*,12*R*,13*S*,14*R*,17*R*)-6-chloro-3(4),13(14),-diepoxy-2,9-diacetoxy-12-isovaleryloxy-8-hydroxybriaran-5(16)-dien-18,7-olide.

**Figure 5:**
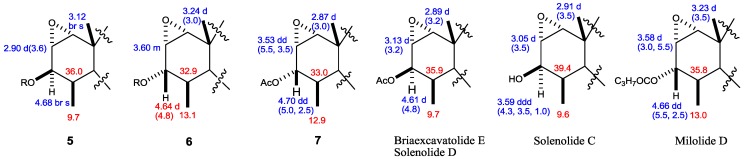
Selected ^1^H- and ^13^C-NMR data of Compounds **5**–**7** and compared with known compounds.

The HRESIMS of briaviolide F (**6**) showed a *pseudo*-molecular ion peak at *m/z* 593.2125 [M + Na]^+^, consistent with a molecular formula of C_28_H_39_O_10_Cl (Δ = 10). Cross comparison of ^1^H- and ^13^C-NMR spectroscopic data of **6** (Table 1 and Table 2) with those of **5** revealed that the differences were the substituents at C-2 and configurations at C-12. Comparison of the proton chemical shift of H-2 with that of milolide D [23] (Figure 5) could confirm the presence of a hydroxyl group at C-2 (δ_C_ 74.0) in **6**. Signals of an acetyl group (δ_H_ 2.20; δ_C_ 169.8, 22.0) located at C-9 (δ_C_ 68.7) and one hexanoate group (δ_H_ 2.28, 1.61, 1.31, 1.31, 0.90; δ_C_ 173.1, 34.0, 24.8, 31.4, 22.4, 14.0) located at C-12 (δ_C_ 69.7) were determined by their HMBC correlations (Figure 2). The NOESY correlations (Figure 4) of H-10/H-2 and H-9, H-9/Me-19 suggested that H-2, H-9, H-10 and Me-19 were placed in α-orientation. Correlations of H-7/H-6 and H-17, H-12/H-13 and Me-20, H-14/H-13 and Me-15 agreed with β-orientations for all these groups. Thus, Compound **6** was established as a 2β-hydroxyl-12α-hexanoyl derivative of **5**.

Briaviolide G (**7**) had the molecular formula C_26_H_33_O_11_Cl, as determined by HRESIMS analysis. It was found that the ^1^H-, ^13^C-NMR (Table 1 and Table 2) and IR spectroscopic data were very similar to those of Compound **6**. However, the hexanoate group at C-12 in **6** was replaced by an acetyl group at C-2 in **7**. This finding was supported by the HMBC correlations between H-2 (δ_H_ 5.23, d, *J* = 9.5 Hz)/carbonyl carbon (δ_C_ 170.0) and H-12 (δ_H_ 4.70, dd, *J* = 5.0, 2.5 Hz)/carbonyl carbon (δ_C_ 169.4). Comparing the ^1^H- and ^13^C-NMR data of **7** with those of solenolide D [25] and briaexcavatolide E [22] indicated their resemblance, except for the chemical shifts around C-12 and C-13. Based on Kobayashi’s and Williams’s study [26], the configuration of 12-OH could be assigned (Figure 5). By comparison of the coupling constant of H-12/H-13 and the chemical shift of C-20, as well as the NOESY correlation between H-12 and Me-20 (δ_H_ 1.06), the acetyl group at C-12 was assigned to be α-face. The other NOSEY correlations (Figure 4) also indicated that Compound **7** had identical configurations as those of **6**. Therefore, **7** was assigned a 2β-acetoxyl-12α-acetoxyl derivative of Compound **6**.

Briaviolide H (**8**) was obtained as an amorphous gum. The molecular formula of **8** was determined as C_24_H_34_O_9_ by its HRESIMS. The IR spectrum showed absorption bands due to hydroxyl group (3440 cm^−1^), γ-lactone (1732 cm^−1^) and ester carbonyl (1712 cm^−1^). The ^1^H- and ^13^C-NMR spectra of **8** in CDCl_3_ gave broad signals, while those in acetone*-d*_6_ were well resolved. Characteristic resonances due to three methyl protons (δ_H_ 1.02, d, *J* = 7.2 Hz; 1.08, s; 1.85, s; each 3H) and two acetyl protons (δ_H_ 1.90, s, 6H) were observed in the ^1^H-NMR spectrum (Table 1). Signals of six oxygenated carbons (δ_C_ 74.8, 79.9, 83.6, 75.7, 69.4, 75.7), two acetyl carbons (δ_C_ 168.6 and 169.0) and a γ-lactone carbonyl carbon (δ_C_ 175.1) were observed in the ^13^C-NMR spectrum (Table 2). The HMBC correlations of H-2 (δ_H_ 5.34, d, *J* = 9.2 Hz)/carbonyl carbon (δ_C_ 168.6) and H-14 (δ_H_ 4.68, br s)/carbonyl carbon (δ_C_ 169.0) helped to locate two acetoxyl groups at C-2 and C-14. The above data suggested that Compound **8** is a highly oxygenated 8-hydroxybriarane-type diterpenoid. The ^1^H-NMR spectrum of **8** was similar to that of frajunolide A [19], except that the chemical shift of H-12 (δ_H_ 4.13, dd, *J* = 10.8, 5.2 Hz; in CDCl_3_) was located upfield as compared with frajunolide A (δ_H_ 5.35). The last remaining oxygen can only be accounted for by a hydroxyl group attached on C-9, because the chemical shift of H-9 (δ_H_ 4.68, br s) was also shifted upfield in comparison with frajunolide A (δ_H_ 5.37). The above data combined with HMBC correlations (Figure 2) revealed that 9- and 12-acetyl groups in frajunolide A were replaced by hydroxyl groups in **8**. The NOESY correlations of H-2/H-10, H-10/H-9 and Me-19 in **8** suggested that the configurations of them were α-oriented. On the other hand, the correlations of H-6/H-7, H-7/H-17, H-14/Me-15 agreed with a β-configuration of H-7, H-14, Me-15 and H-17. The large coupling constant (*J*_6,7_ = 9.6 Hz) confirmed the anti-parallel arrangement of H-6 and H-7 and the β-orientation of H-7 [19]. It was concluded that briaviolide H (**8**) has the structure of (1*S*,2*S*,6*Z*,7*S*,8*R*,9*S*,10*S*,12*S*,14*S*,17*R*)-2,14-diacetoxy-12-hydroxy-8,9-dihydroxybriaran-5(6)-dien-18,7-olide.

The molecular formula C_24_H_34_O_10_ was assigned to Compound **9** from its HRESIMS and ^13^C NMR data (Table 2). The spectroscopic values of **9** suggested a briarane structure similar to that of **8** with one additional hydroperoxy group at C-12 (δ_C_ 83.8). The configuration of Compound **9** was further determined by the NOESY experiments (Figure 4), and the correlations revealed that **9** possessed the same relative configurations as those of **8**. Thus, briaviolide I (**9**) was assigned as 12-hydroperoxyl derivative of Compound **8**.

Briaviolide J (**10**) had the molecular formula C_26_H_36_O_10_, as determined by its HRESIMS and DEPT ^13^C NMR data. The IR absorptions of **10** were found at 3426, 1732 and 1675 cm^−1^, which indicated the presence of hydroxyl, a γ-lactone and ester groups. The ^1^H- and ^13^C-NMR data (Table 1 and Table 2) revealed that **10** was an 8-hydroxybriarane-type diterpenoid and was structurally similar to **8** and **9**. Comparisons of their NMR and MS data showed that the only difference between **8** and **10** was the presence of an acetate group at C-12 in **10**. Acetylation of **8** afforded a product identical to Compound **10**. Thus, it was concluded that **10** is 12-acetoxyl derivative of Compound **8**.

In addition, six known briaranes, solenolides A and D [25], excavatolide A [26], briaexcavatolide I [22], 4β-acetoxy-9-deacetystylatulide lactone and 9-deacetylstylatulide lactone [27], were identified. The new isolated briaranes **1**–**10** and derivative **11** were evaluated for anti-inflammatory activities on superoxide-anion generation and elastase release by human neutrophils in response to *N*-formyl-methionyl-leucyl-phenylalanine (fMLP)/Cytochalasin B (CB). The inhibition percentages of these compounds at the concentration of 10 μg/mL are summarized in Table 3. The bioassay data showed that Compounds **5** and **9** have moderate activities on both of superoxide-anion generation and elastase release, while Compound **11** has selective activity on the inhibition of elastase release.

**Table 3:** Inhibitory effects of Compounds **1**–**11** on superoxide anion generation and elastase release by human neutrophils in response to fMLP/CB.

Compound	Inhibition (%) ^a^	
	Superoxide Anion	Elastase Release
**1**	6.09 ±1.40 *	11.04 ± 7.22
**2**	6.43 ± 2.17 *	13.43 ± 2.66 **
**3**	16.87 ± 4.86 *	6.40 ± 4.29
**4**	4.48 ± 1.47 *	9.31 ± 6.64
**5**	34.17 ± 0.79 ***	26.03 ± 9.51
**6**	17.35 ± 6.91	14.34 ± 5.28
**7**	3.25 ± 2.35	16.66 ± 3.12 **
**8**	6.01 ± 4.16	18.78 ± 2.29 **
**9**	28.66 ± 1.99 ***	28.81 ± 6.37 *
**10**	11.64 ± 3.92 *	14.62 ± 4.41 **
**11**	6.09 ± 4.09 **	28.60 ± 7.54 *
genistein ^b^	65.05 ± 6.12	52.45 ± 6.34

^a^ At a concentration of 10 μg/mL for each compound. Results are presented as the mean ± SEM (*n* = 3). * *p* < 0.05, ** *p* < 0.01, *** *p* < 0.001 compared with the control value; ^b^ Positive control.

## 3. Experimental Section

### 3.1. General Experimental Procedures

The melting point was measured on a BÜCHI Melting Point B-540 apparatus (Buchi, Flawil, Switzerland) and uncorrected. Optical rotations were recorded on a JASCO DIP-1020 polarimeter (Jasco, Tokyo, Japan). IR spectra were measured on a JASCO FT/IR-4100 spectrophotometer (Jasco, Tokyo, Japan). HR-ESI-MS were taken on a JEOL JMS-HX 110 mass spectrometer (Jeol, Tokyo, Japan). The NMR spectra were recorded either on a Bruker Avance 300, or a 400 NMR spectrometer, or on a Varian MR 400 NMR spectrometer (Varian, Santa Clara, CA, USA). The chemical shifts were given in δ (ppm) and coupling constants in Hz. Silica gel 60 (Merck, Darmstadt, Germany) was used for column chromatography (CC), and pre-coated silica gel plates (Merck, Darmstadt, Germany, Kieselgel 60 F-254, 1 mm) were used for preparative TLC. Sephadex LH-20 (Amersham Pharmacia Biotech AB, Sweden) was used for separation. LiChrospher^®^ Si 60 (5 μm, 250–10, Merck, Darmstadt, Germany) and LiChrospher^®^ 100 RP-18e (5 μm, 250–10, Merck, Darmstadt, Germany) were used for NP-HPLC and RP-HPLC (Merck, Darmstadt, Germany), respectively.

### 3.2. Animal Material

The gorgonian, *Briareum violacea* (Quoy and Gaimard), was collected in Pingtong County of southern Taiwan by scuba diving at a depth of 15 m, in May 2007. The fresh gorgonian was immediately frozen after collection and kept at −20 °C until being processed. A voucher specimen was deposited in the School of Pharmacy, College of Medicine, National Taiwan University.

### 3.3. Extraction and Isolation

The gorgonian, *B. violacea* (wet weight, 2.5 kg), was minced and extracted with acetone/MeOH (3 × 5 L) at room temperature, and the extracts were combined and concentrated under vacuum. The dark green crude residue was partitioned between EtOAc and H_2_O (1:1). The EtOAc-soluble portion was shaken with *n*-hexane/MeOH/H_2_O (4:3:1), and the MeOH layer (15 g) was evaporated and separated on Sephadex LH-20 to give eight fractions (L1 to L8). Fraction L3 (9.5 g) was subjected to flash column chromatography using silica gel and a gradient of *n*-hexane/EtOAc/MeOH to obtain 25 fractions (L3-1 to L3-25). Crystallization of L3-16 (*n*-hexane/EtOAc, 2:1; 640 mg) furnished 9-deacetylstylatulide lactone (123 mg). The MeOH-soluble portion of fraction L3-16 was separated on C_18_ reversed-phase (RP) HPLC using MeOH/H_2_O/CH_3_CN (50:45:10) to yield Compound **5** (9 mg), excavatolide A (6.5 mg) and stylatulide lactone (2 mg). Fraction L3-17 (*n*-hexane/EtOAc, 3:2; 1.6 g) was separated by RP-HPLC using MeOH/H_2_O/CH_3_CN (50:50:5) to afford Compound **6** (8.7 mg). Fraction L3-18 (*n*-hexane/EtOAc, 1:1; 2.0 g) was subjected to column chromatography using silica gel and a gradient of *n*-hexane/EtOAc/MeOH to obtain 10 fractions (L3-18-1 to L3-18-10). Fraction L3-18-2 (185 mg) was subjected to RP-HPLC using MeOH/H_2_O/CH_3_CN (65:30:5) to give Compounds **2** (3.5 mg), **3** (3.8 mg) and solenolide A (7.8 mg). Fraction L3-18-4 (231 mg) was separated by RP-HPLC using MeOH/H_2_O/CH_3_CN (65:30:5) to afford Compound **8** (11.0 mg) and solenolide D (9.4 mg). Fraction L3-18-7 was subjected on RP-HPLC using MeOH/H_2_O/CH_3_CN (55:45:5) to obtain Compounds **1** (12 mg), **7** (28 mg) and briaexcavatolide I (5.4 mg). Fraction L3-18-8 (64 mg) was purified by RP-HPLC using MeOH/H_2_O/CH_3_CN (65:30:5) to yield Compound **10** (10.5 mg). Fraction L3-20 (*n*-hexane/EtOAc, 1:2; 300 mg) was purified by RP-HPLC to afford Compounds **4** (3.5 mg) and **9** (4.3 mg).

Briaviolide A (**1**): Colorless amorphous prism; mp. 174–175 °C; ${\left[\text{α}\right]}_{\text{D}}^{24}$ −79 (*c* 0.5, CH_2_Cl_2_); IR ν_max_ 3524, 3365, 2980, 2949, 1767, 1737, 1375, 1231 cm^−1^; ^1^H-NMR data (300 MHz, CDCl_3_), see Table 1; ^13^C-NMR data (75 MHz, CDCl_3_), see Table 2; ESIMS *m/z* 521 [M + Na]^+^, 523 [M + Na + 2]^+^; HRESIMS *m/z* 521.1554 [M + Na]^+^ (calcd. for C_2__4_H_3__1_^35^ClO_9_Na, 521.1557).

Briaviolide B (**2**): Colorless amorphous gum; ${\left[\text{α}\right]}_{\text{D}}^{24}$ −45 (*c* 0.1, CH_2_Cl_2_); IR ν_max_ 3453, 2965, 2942, 1783, 1738, 1732, 1278 cm^−1^; ^1^H-NMR data (400 MHz, CDCl_3_), see Table 1; ^13^C-NMR data (100 MHz, CDCl_3_), see Table 2; ESIMS *m/z* 577 [M + Na]^+^, 579 [M + Na + 2]^+^; HRESIMS *m/z* 577.1812 [M + Na]^+^ (calcd. for C_27_H_35_^35^ClO_10_Na, 577.1816).

Briaviolide C (**3**): Colorless powder; ${\left[\text{α}\right]}_{\text{D}}^{24}$ −6 (*c* 0.1, CH_2_Cl_2_); IR ν_max_ 3429, 2965, 2928, 1776, 1738, 1373, 1213 cm^−1^; ^1^H-NMR data (400 MHz, CDCl_3_), see Table 1; ^13^C-NMR data (100 MHz, CDCl_3_), see Table 2; ESIMS *m/z* 587 [M + Na]^+^; HRESIMS *m/z* 587.2101 [M + Na]^+^ (calcd. for C_28_H_36_O_12_Na, 587.2104).

Briaviolide D (**4**): Colorless amorphous gum; ${\left[\text{α}\right]}_{\text{D}}^{24}$ −11 (*c* 0.4, CH_2_Cl_2_); IR ν_max_ 3433, 3015, 2989, 2938, 1776, 1734, 1373, 1220, 1019, 756 cm^−1^; ^1^H-NMR data (400 MHz, CDCl_3_), see Table 1; ^13^C-NMR data (100 MHz, CDCl_3_), see Table 2; ESIMS *m/z* 537 [M + Na]^+^, 539 [M + Na + 2]^+^; HRESIMS *m/z* 537.1506 [M + Na]^+^ (calcd. for C_2__4_H_31_^35^ClO_1__0_Na, 537.1503).

Briaviolide E (**5**): Colorless amorphous powder; ${\left[\text{α}\right]}_{\text{D}}^{24}$ −6 (*c* 0.1, CH_2_Cl_2_); IR ν_max_ 3429, 2965, 2928, 1779, 1738, 1373, 1213 cm^−1^; ^1^H-NMR data (400 MHz, CDCl_3_), see Table 1; ^13^C-NMR data (100 MHz, CDCl_3_), see Table 2; ESIMS *m/z* 612 [M + Na]^+^, 614 [M + Na + 2]^+^; HRESIMS *m/z* 612.2075 [M + Na]^+^ (calcd. for C_2__9_H_3__9_^35^ClO_1__1_Na, 612.2079).

Briaviolide F (**6**): Colorless amorphous gum; ${\left[\text{α}\right]}_{\text{D}}^{24}$ +16 (*c* 0.4, CH_2_Cl_2_); IR ν_max_ 3410, 2981, 2938, 1768, 1742, 1369, 1216, 1019, 756 cm^−1^; ^1^H-NMR data (300 MHz, CDCl_3_), see Table 1; ^13^C-NMR data (75 MHz, CDCl_3_), see Table 2; ESIMS *m/z* 593 [M + Na]^+^, 595 [M + Na + 2]^+^; HRESIMS *m/z* 593.2125 [M + Na]^+^ (calcd. for C_2__8_H_39_^35^ClO_10_Na, 593.2129).

Briaviolide G (**7**): Colorless amorphous gum; ${\left[\text{α}\right]}_{\text{D}}^{24}$ −18 (*c* 0.6, CH_2_Cl_2_); IR ν_max_ 3544, 3447, 2981, 2937, 1782, 1739, 1375, 1223, 1021, 737 cm^−1^; ^1^H-NMR data (400 MHz, CDCl_3_), see Table 1; ^13^C-NMR data (100 MHz, CDCl_3_), see Table 2; ESIMS *m/z* 579 [M + Na]^+^, 581 [M + Na + 2]^+^; HRESIMS *m/z* 579.1605 [M + Na]^+^ (calcd. for C_26_H_33_^35^ClO_11_Na, 579.1609).

Briaviolide H (**8**): Colorless amorphous gum; ${\left[\text{α}\right]}_{\text{D}}^{24}$ +53 (*c* 0.2, CH_2_Cl_2_); IR ν_max_ 3440, 2923, 1732, 1712, 1644, 1375, 1260, 1034, 953, 733 cm^−1^; ^1^H-NMR data (400 MHz, Acetone*-d*_6_), see Table 1; ^13^C-NMR data (100 MHz, Acetone*-d*_6_), see Table 2; ESIMS *m/z* 489 [M + Na]^+^; HRESIMS *m/z* 489.2106 [M + Na]^+^ (calcd. for C_24_H_3__4_O_9_Na, 489.2101).

Briaviolide I (**9**): Colorless amorphous powder; ${\left[\text{α}\right]}_{\text{D}}^{24}$ +20 (*c* 0.4, CH_2_Cl_2_); IR ν_max_ 3425, 2928, 1738, 1715, 1373, 1259 cm^−1^; ^1^H-NMR data (400 MHz, CD_3_OD), see Table 1; ^13^C-NMR data (100 MHz, CD_3_OD), see Table 2; ESIMS *m/z* 505 [M + Na]^+^; HRESIMS *m/z* 505.2046 [M + Na]^+^ (calcd. for C_24_H_34_O_10_Na, 505.2050).

Briaviolide J (**10**): Colorless amorphous gum; ${\left[\text{α}\right]}_{\text{D}}^{24}$ +50 (*c* 0.1, CH_2_Cl_2_); IR ν_max_ 3426, 2927, 2853, 1732, 1675, 1375, 1257, 1044, 737 cm^−1^; ^1^H-NMR data (400 MHz, pyridine-* d*_5_), see Table 1; ^13^C-NMR data (100 MHz, pyridine*-d*_5_), see Table 2; ESIMS *m/z* 531 [M + Na]^+^; HRESIMS *m/z* 531.2204 [M + Na]^+^ (calcd. for C_26_H_36_O_1__0_Na, 531.2206).

Benzyl briaviolide A (**11**): Colorless amorphous gum; ${\left[\text{α}\right]}_{\text{D}}^{24}$ −21 (*c* 0.2, CH_2_Cl_2_); IR ν_max_ 3463, 2985, 2938, 1779, 1734, 1270, 1224 cm^−1^; ^1^H-NMR data (300 MHz, CDCl_3_), see Table 1; ^13^C-NMR data (75 MHz, CDCl_3_); see Table 2; ESIMS *m/z* 625 [M + Na]^+^, 627 [M + Na + 2]^+^; HRESIMS *m/z* 625.1820 [M + Na]^+^ (calcd. for C_31_H_35_^35^ClO_1__0_Na, 625.1816).

### 3.4. Benzoylation of Briaviolide (**1**)

Compound **1** (3.0 mg) was stirred with 0.1 mL of benzoyl chloride in pyridine (1.0 mL) for 20 h at room temperature. After evaporation, the residue was separated by a C_18_ reversed-phase HPLC column (MeOH/H_2_O, 4:1) to give pure Compound **11** (2.8 mg).

### 3.5. Single Crystal X-Ray Structure Determination of Briaviolide (**1**)

A suitable colorless crystal (0.20 × 0.15 × 0.10 mm^3^) of **1** for diffraction was obtained by simple evaporation from methanol solution. Crystal data: C_2__4_H_3__1_ClO_9_, orthorhombic, *a* = 10.4634(2) Å, *b* = 13.9191(3) Å, *c* =17.1825(3) Å, *V* = 2502.48(8) Å^3^, space group P2_1_2_1_2_1_, *Z* = 4, D_calcd_ = 1.324 Mg/m^3^, λ= 1.54178 Å, μ(Mo Kα) 1.783 mm^−1^, F(000) = 1056, T = 295(2) K. A total of 17,948 reflections were collected, of which 4536 unique reflections (*R*_int_ = 0.0427) with I > 2σ(*I*) were used for the analysis. The data was solved using the direct method, and the structure was refined by a full-matrix least-squares procedure on F^2^ values. All non-hydrogen atoms were refined with anisotropic thermal parameters. The hydrogen atom positions were geometrically idealized and allowed to ride on their parent atoms. The final indices were R1 0.0499, wR2 0.1420 with goodness-of-fit = 1.149. The final X-ray molecular model is shown in Figure 3b.

### 3.6. Anti-Inflammatory Assays

#### 3.6.1. Human Neutrophils Elastase Release

Degranulation of azurophilic granules was determined by elastase release, as described previously [28]. Experiments were performed using MeO-Suc-Ala-Ala-Pro-Val-*p*-nitroanilide as the elastase substrate. After supplementation with MeO-Suc-Ala-Ala-Pro-Val-*p*-nitroanilide (100 μM), neutrophils (6 × 10^5^ cell/mL) were equilibrated at 37 °C for 2 min and incubated with each test compound for 5 min. Cells were activated by fMLP (100 nM)/CB (0.5 μg/mL), and changes in absorbance at 405 nm were monitored continuously for elastase release. The results are expressed as the percentage of the initial rate of elastase release in the fMLP/CB-activated, test compound-free (DMSO) control system.

#### 3.6.2. Human Neutrophil Superoxide Generation

Human neutrophils were obtained by means of dextran sedimentation and Ficoll centrifugation. Superoxide anion production was assayed by monitoring the superoxide dismutase-inhibitable reduction of ferricytochrome *c* [29]. In brief, after supplementation with 0.5 mg/mL ferricytochrome *c* and 1.0 mM Ca^2+^, neutrophils were equilibrated at 37 °C for 2 min and incubated with drugs for 5 min. Cells were activated with 100 nM fMLP for 10 min. When fMLP was used as a stimulant, CB (1 μg/mL) was incubated for 3 min before activation by the peptide (fMLP/CB). Changes in absorbance with the reduction of ferricytochrome *c* at 550 nm were continuously monitored in a double-beam, six-cell positioner spectrophotometer with constant stirring (Hitachi U-3010, Tokyo, Japan). Calculations were based on differences in the reactions with and without SOD (100 U/mL) divided by the extinction coefficient for the reduction of ferricytochrome *c*.

## 4. Conclusions

Sixteen briarane diterpenoids, including ten new compounds briaviolides, A–J (**1**–**10**), were successfully isolated from the Taiwanese soft coral, *Briareum violacea*, and their structures determined. The inhibitory effects of the isolates and new derivative **11** on superoxide-anion generation and elastase release by human neutrophils in response to fMLP/CB were evaluated. Compounds **5** and **9** showed moderate anti-inflammatory activities at a concentration of 10 μg/mL. Compound **11**, derived from Compound **1**, showed better inhibition of elastase release than that of **1**. Further comparison of the activities of those compounds may suggest that β-orientation and the chain length of ester groups at C-12 are important for the anti-inflammatory activities in briarane-type diterpenoids.
